# Supplementary figures and images for: In vitro reconstitution reveals membrane clustering and RNA recruitment by the enteroviral AAA+ ATPase 2C
Source: PLoS Pathog. 2024 Aug 5;20(8):e1012388. doi: 10.1371/journal.ppat.1012388 (PMC11326647; doi:10.1371/journal.ppat.1012388)

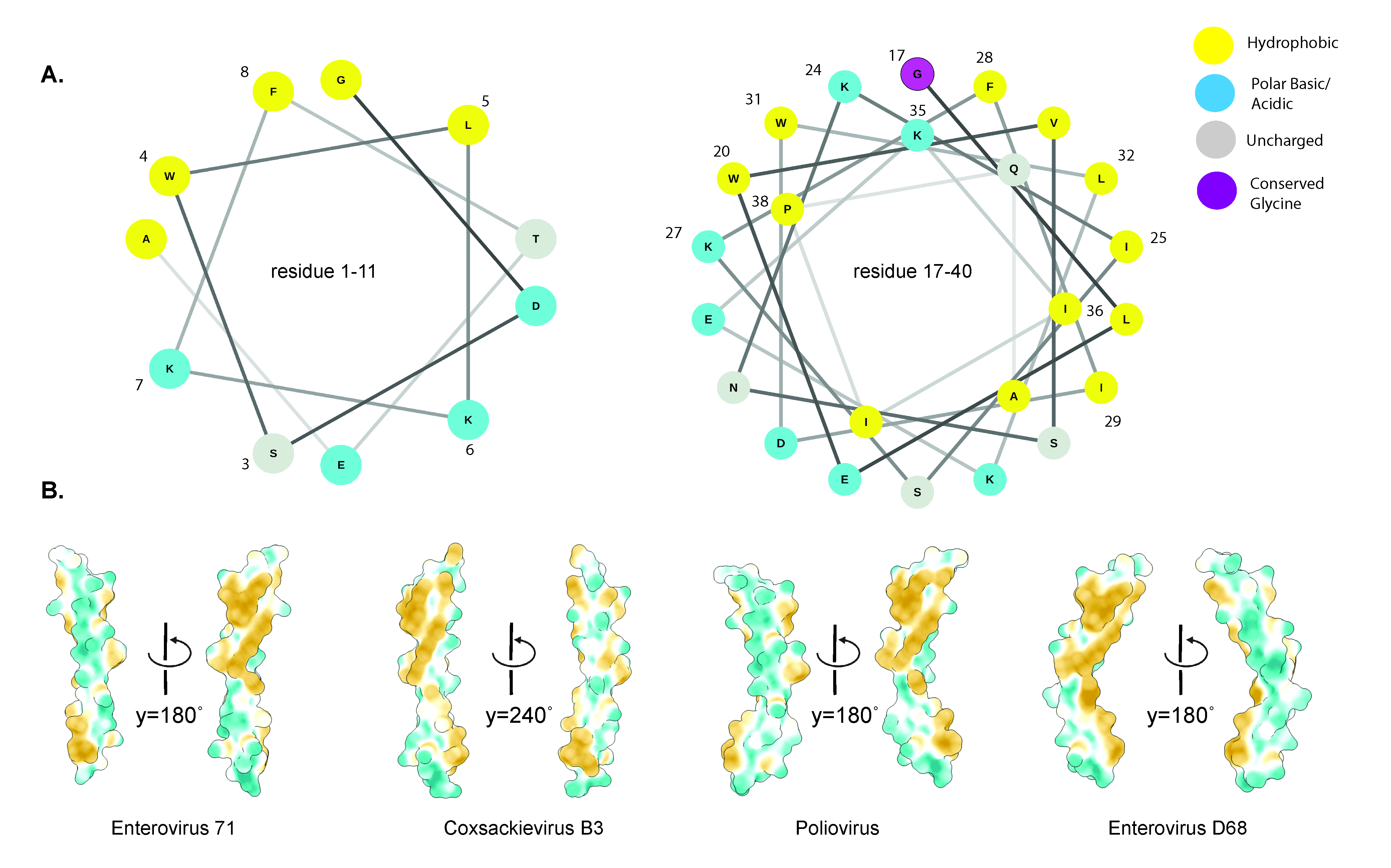

Supplement: S1 Fig — (A) Helical wheel of the AH1 and AH2 regions of poliovirus type 1 Mahoney 2C (GenBank: V01148.1) showing the distribution of hydrophobic and hydrophilic residues. Conserved residues are numbered. (B) Isosurface representations of Alphafold2-predicted structures of the MBD of different enterovirus genotypes showing the distribution of hydrophilic (blue) and hydrophobic (yellow) residues. For each structure prediction, two views related by the indicated rotation are shown. (TIF) [file ppat.1012388.s001.tif]

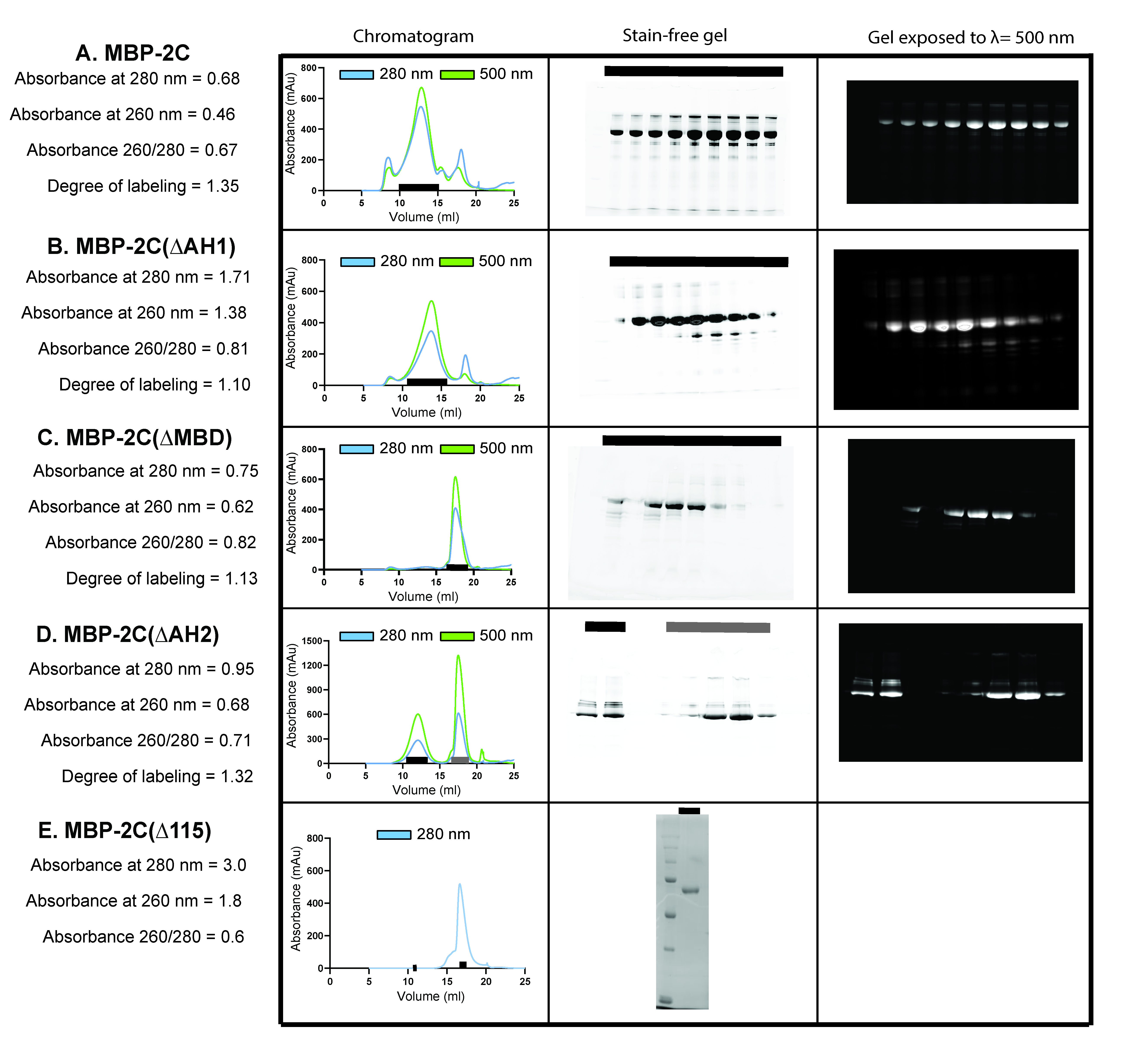

Supplement: S2 Fig — (A-D) Absorbance at 280 nm, 260 nm, 260/280 ratio and degree of labeling for different constructs of MBP tagged 2C and the corresponding chromatogram obtained from Superose 6 Increase. The fractions that were run on an SDS-PAGE gel are marked on the chromatogram as solid lines. The SDS-PAGE gels were both exposed to UV for visualization of protein fractions and at ʎ = 500 nm to excite the ATTO 488 fluorophore to visualize the labeled protein. (E) MBP-2C(Δ115) Absorbance at 280 nm, 260 nm, and 260/280 ratio. The SEC chromatogram obtained from Superose 6 Increase and the corresponding stain free gel of the fraction marked on the chromatogram. (TIF) [file ppat.1012388.s002.tif]

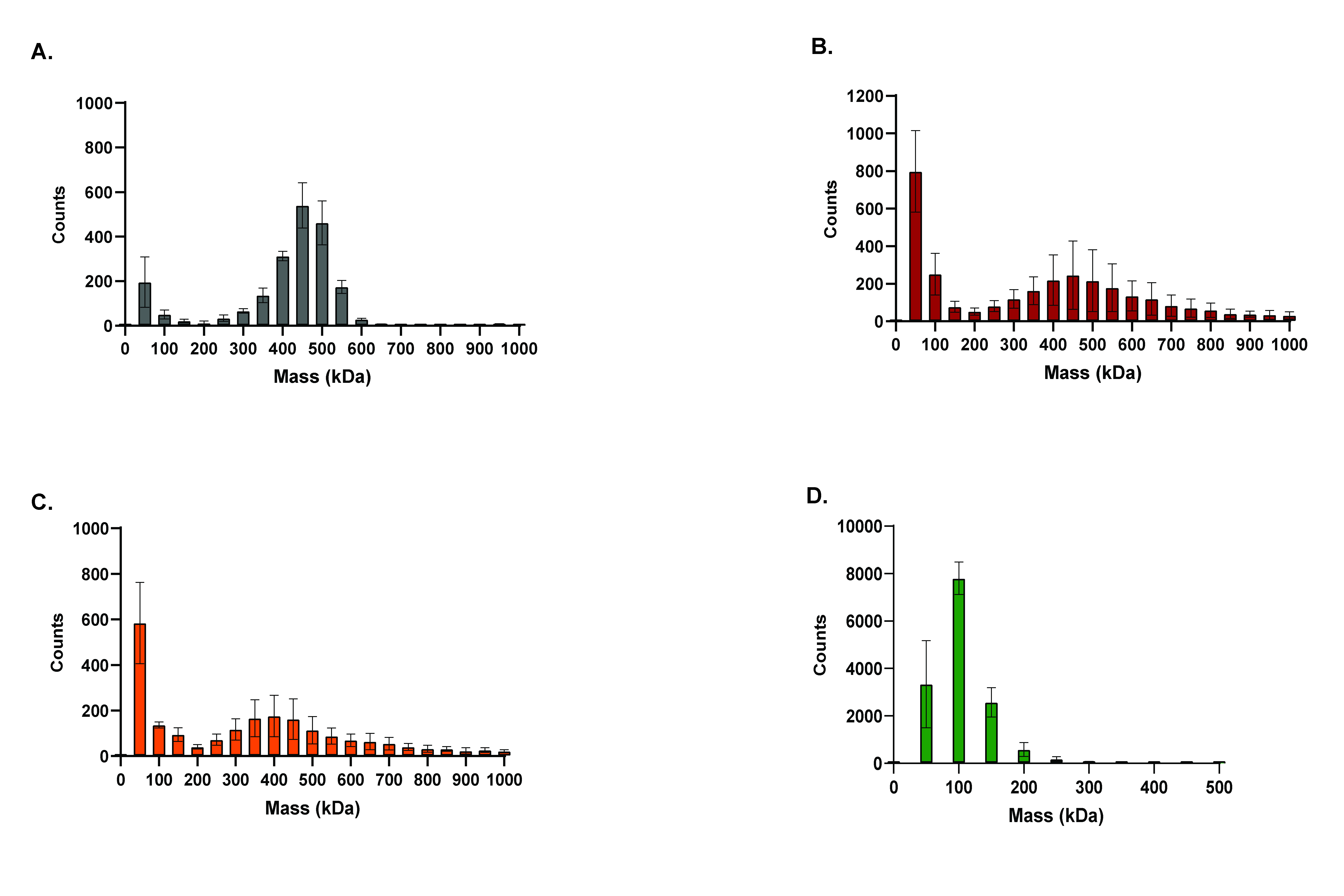

Supplement: S3 Fig — Raw data (mean and standard deviation) from triplicates of mass photometry analysis of 100 nM each of (A) apoferritin, (B) MBP-2C, (C) MBP-(ΔAH1) and (D) MBP-(ΔAH2). (TIF) [file ppat.1012388.s003.tif]

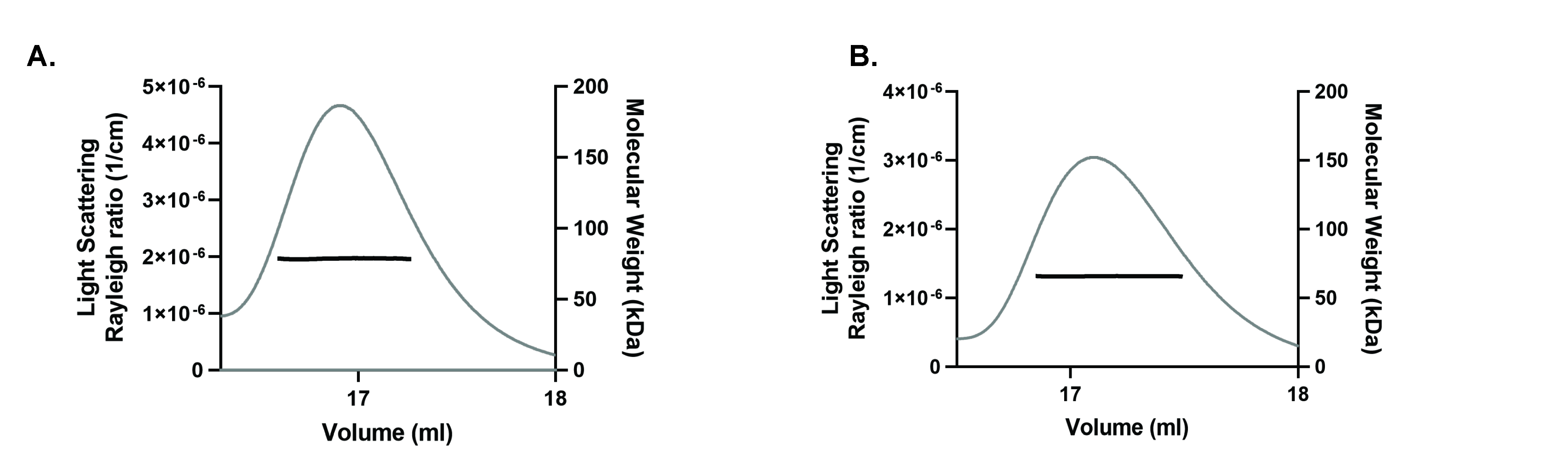

Supplement: S4 Fig — SEC-MALS data showing estimated molecular mass throughout the main elution peak of (A) MBP-2C(ΔMBD) and (B) MBP-2C(Δ115) at concentrations of 38 and 134μM, respectively. The calculated masses are shown as a thick black line. MBP-2C(ΔMBD) had an estimated mass of 78 kDa, whereas MBP-2C(Δ115) had an estimated mass of 66 kDa. (TIF) [file ppat.1012388.s004.tif]

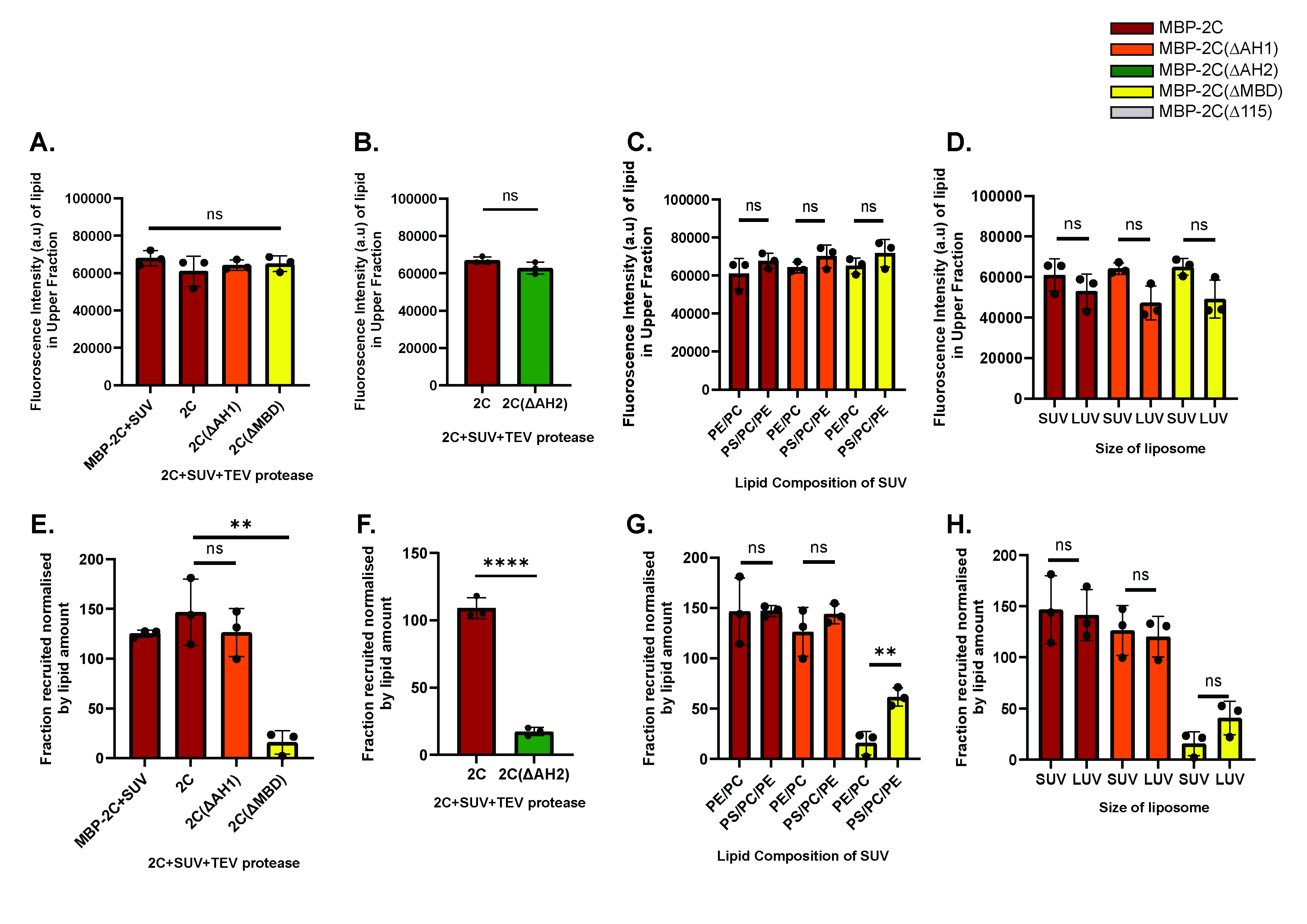

Supplement: S5 Fig — (A) Lane 1: protein ladder. Lanes 2–8: Reactions including MBP-2C or MBP-2C(ΔAH1), and other components as indicated, at the end of the reaction. Lanes 3 and 6: 0.8 μM of the indicated 2C construct. Lanes 4–5 and 7–8: 3 μM of the indicated 2C construct. The two bands observed in the TEV protease prep were both confirmed to be TEV protease by mass spectrometry (S14 Fig). (B) As (A) but with 0.8 μM MBP-2C(ΔMBD) and MBP-2C(ΔAH2), as well as other components as indicated. (TIF) [file ppat.1012388.s005.tif]

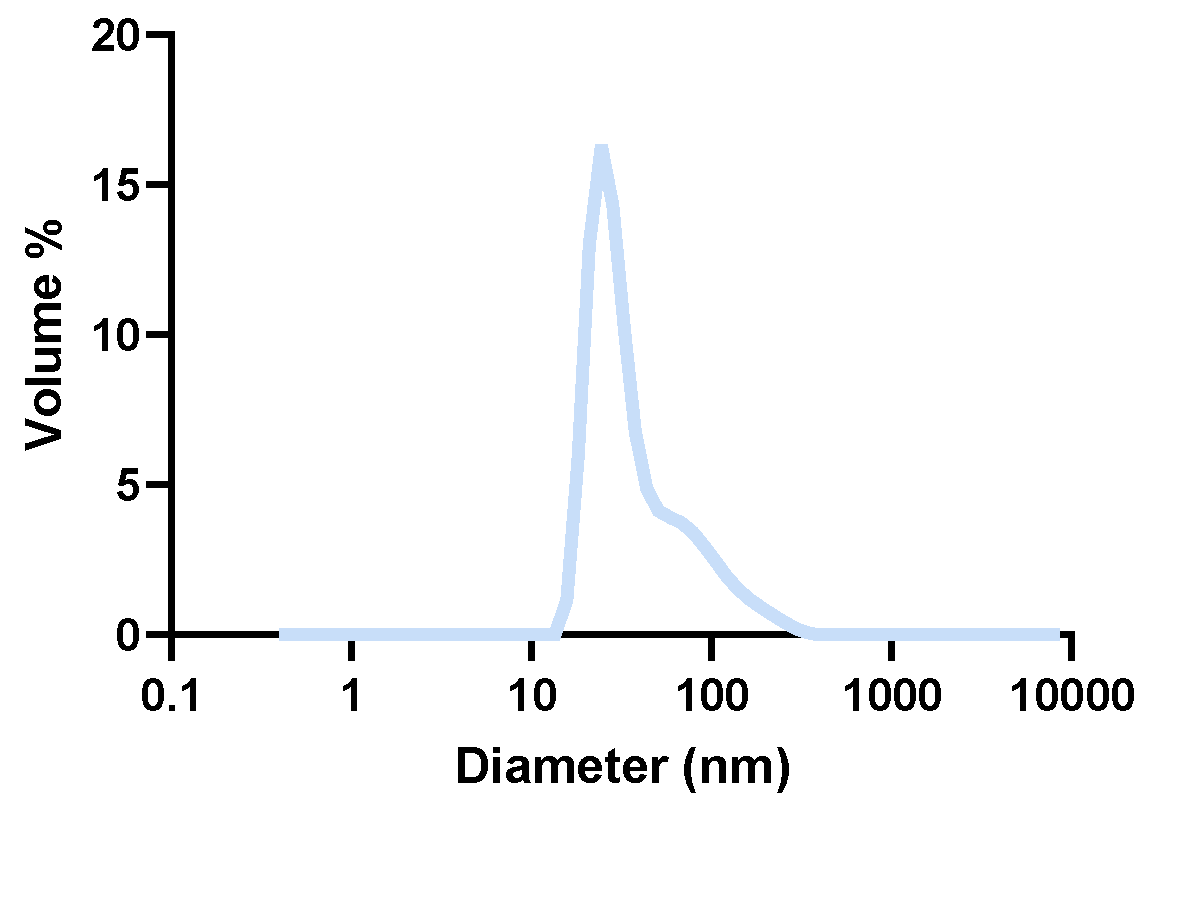

Supplement: S6 Fig — (A-D) Lipid fluorescence signal in the upper fraction of the assays shown in Fig 3C–3F. Error bars represent a standard deviation of three repeats of the experiment. Statistical significance by unpaired two-tailed Student’s t test; ns > 0.05. (E-H) Fraction 2C recruited to the upper fraction, normalized by amount of lipid in the upper fraction, for the assays shown in Fig 3C–3E. Error bars represent a standard deviation of three repeats of the experiment. Statistical significance by unpaired two-tailed Student’s t-test; ns: p>0.05, **: p<0.01. (TIF) [file ppat.1012388.s006.tif]

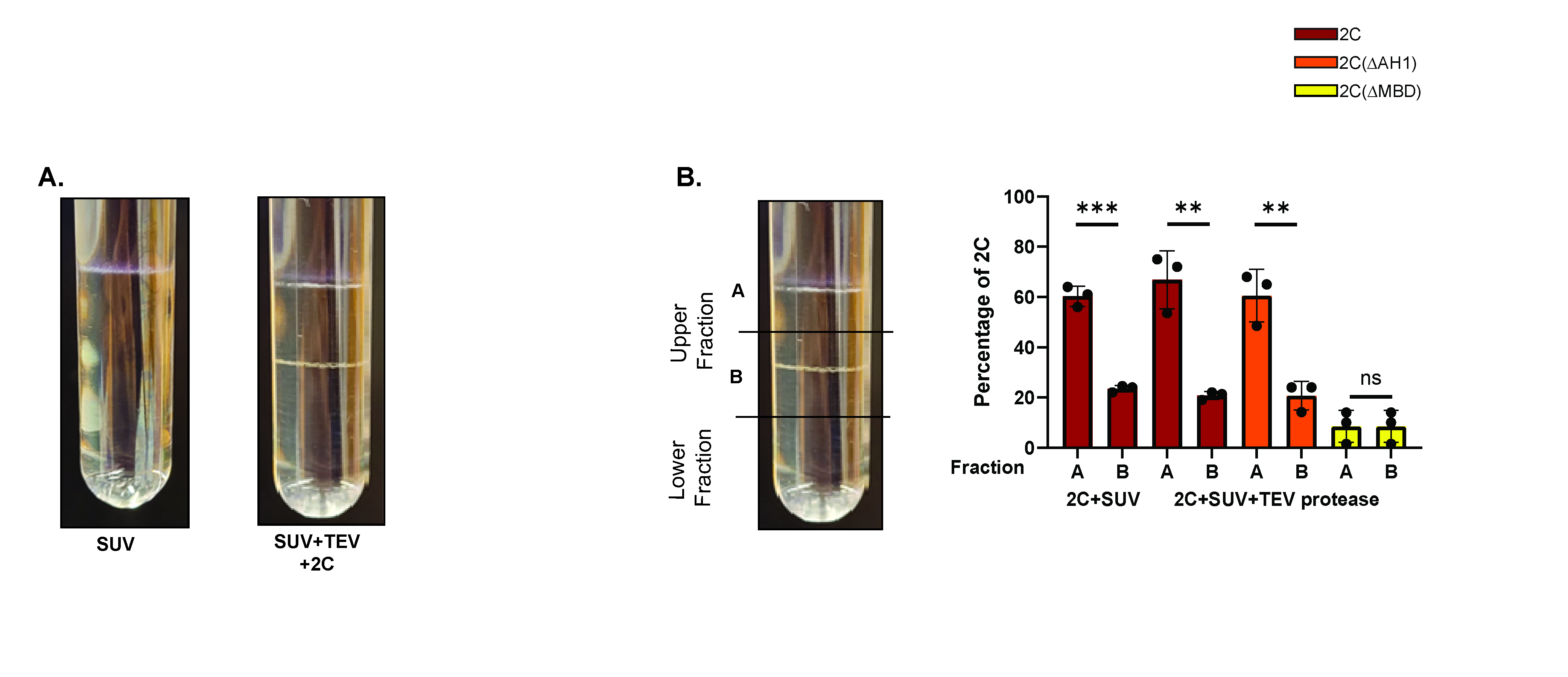

Supplement: S7 Fig — (A) Standard curve with different concentrations of zinc acetate at 202 nm recorded using inductively coupled plasma-optical emission spectrometry (ICP-OES). (B) Emission at 202 nm corresponding to zinc, measured for labeled and unlabeled MBP-2C. Error bars represent a standard deviation of three repeats of the experiment. Statistical significance by unpaired two-tailed Student’s t-test; ns: p>0.05. (TIF) [file ppat.1012388.s007.tif]

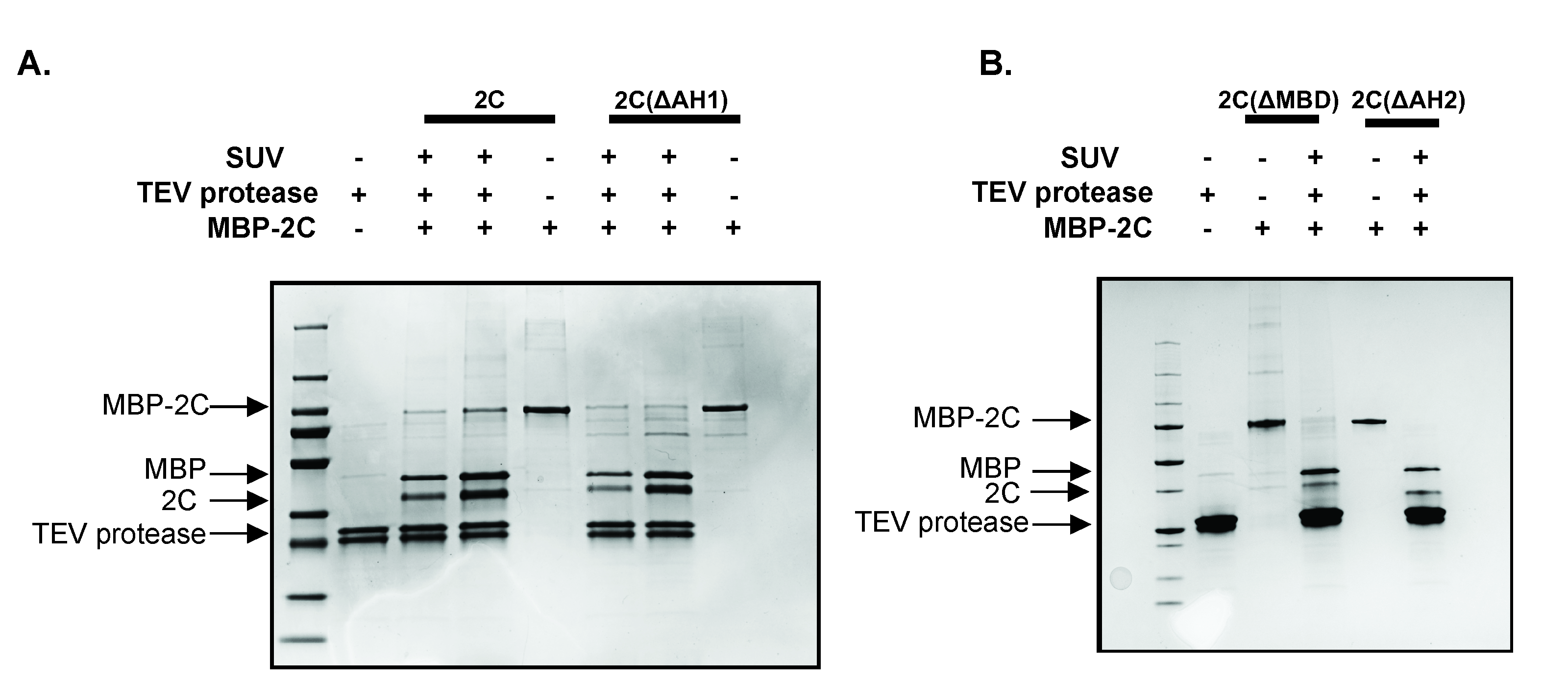

Supplement: S8 Fig — Dynamic light scattering graph showing the size distribution of PE/PC SUVs (size distribution by volume). Average size of the SUVs was calculated to be 30 nm. (TIF) [file ppat.1012388.s008.tif]

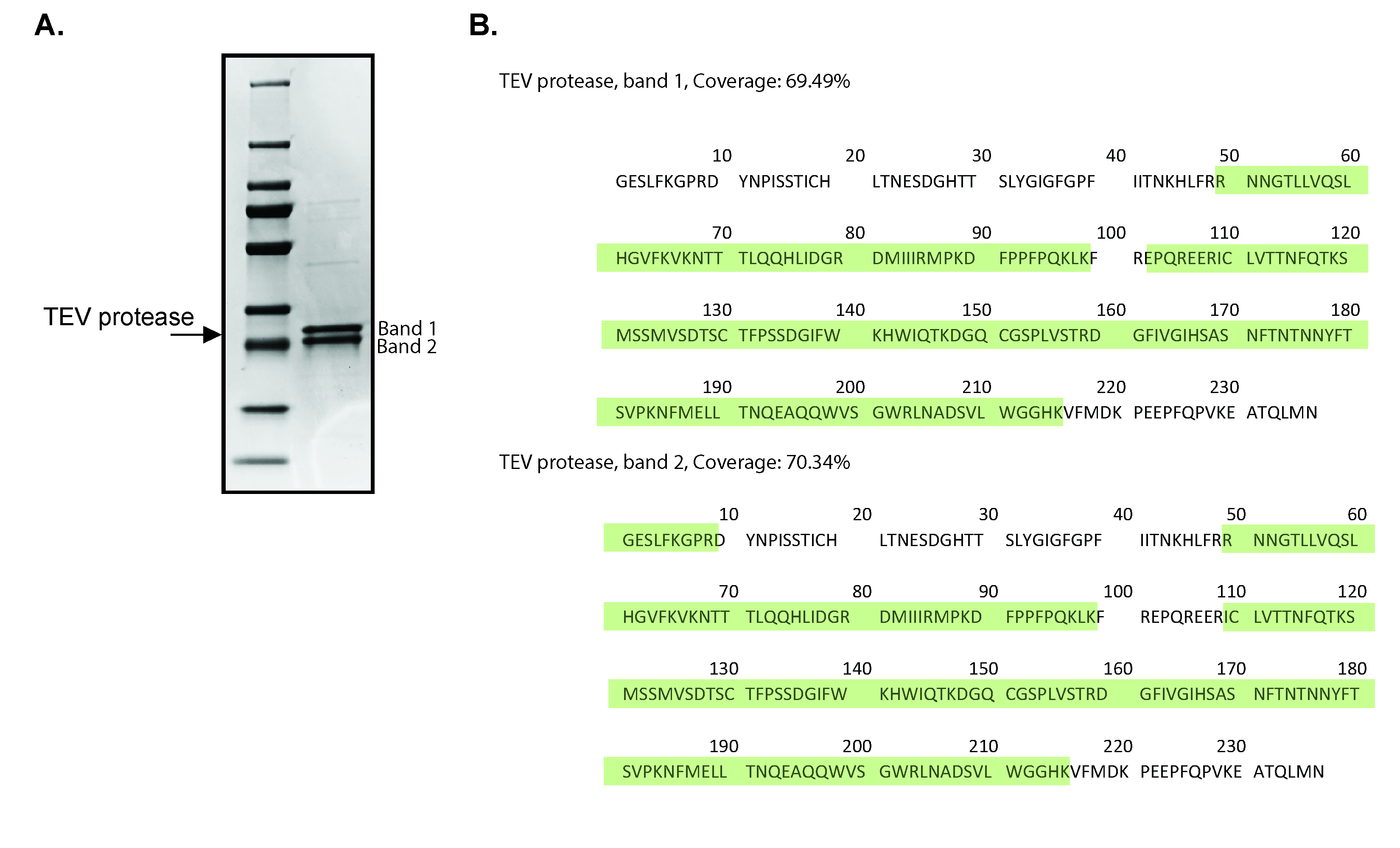

Supplement: S9 Fig — (A) Photograph of representative centrifuge tubes after the flotation assay with SUVs only (single lipid band), and SUVs + TEV protease + MBP-2C (two lipid bands). (B) Photograph of centrifuge tube, indicating the upper fractions A, B, and the lower fraction. The percentage of the total 2C fluorescence in fractions A and B are shown. Error bars represent the standard deviation of three repeats of the experiment. Statistical significance by unpaired two-tailed Student’s t-test; **: p<0.01, ****: p<0.0001. (TIF) [file ppat.1012388.s009.tif]

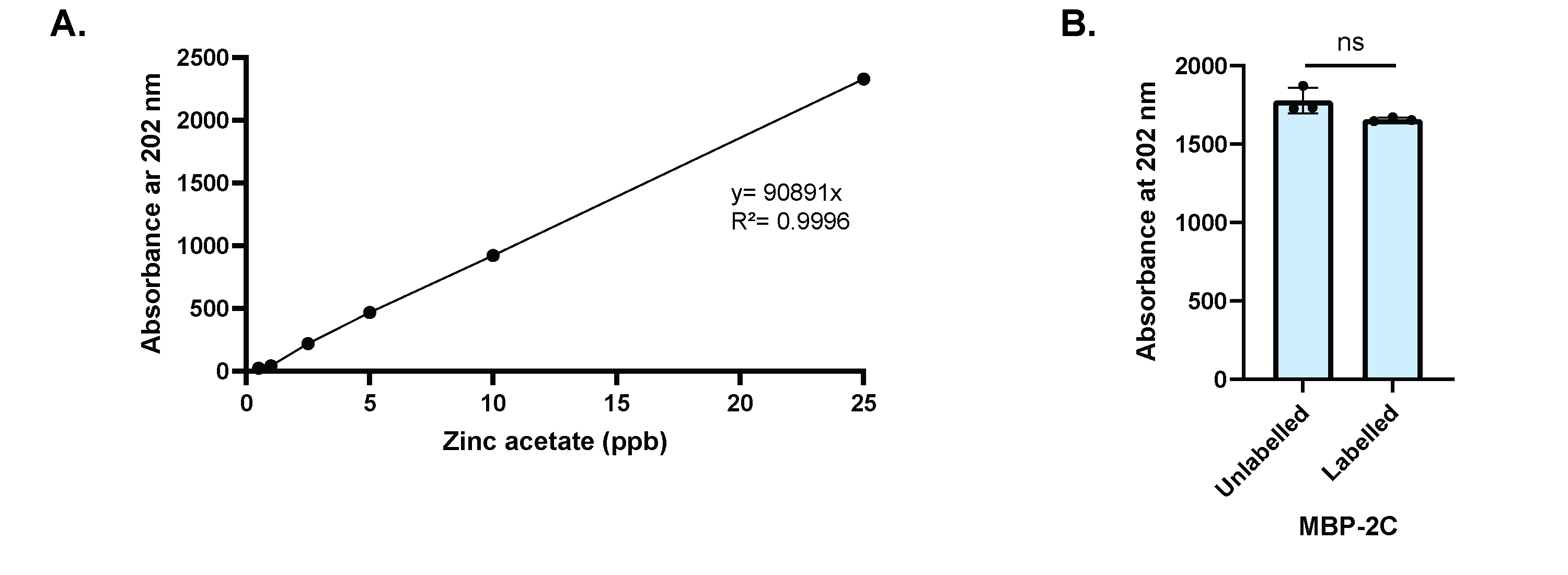

Supplement: S10 Fig — Representative images showing effect of different 2C constructs on SUVs (A) SUV only, (B) SUV+2C (0.8 μM and 3 μM), (C) SUV+2C(ΔAH1) (0.8 μM and 3 μM), (D) SUV+2C(ΔAH1) (0.8 μM), (E) SUV+2C(ΔAH2) (0.8 μM). (TIF) [file ppat.1012388.s010.tif]

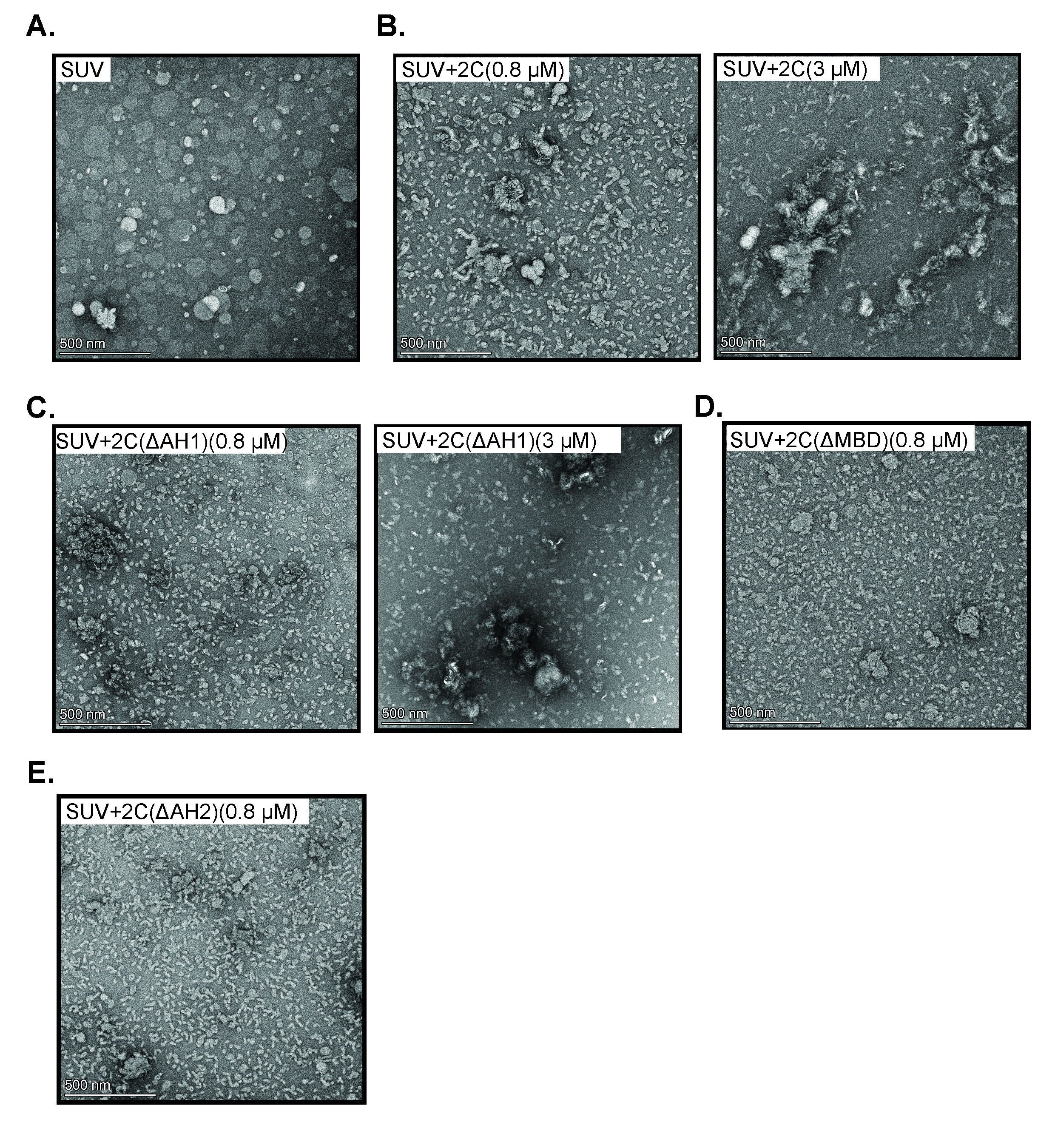

Supplement: S11 Fig — Fluorescence scan of non-denaturing polyacrylamide gel showing 5′TET-labeled ssRNA and 5′TET-labeled dsRNA. (TIF) [file ppat.1012388.s011.tif]

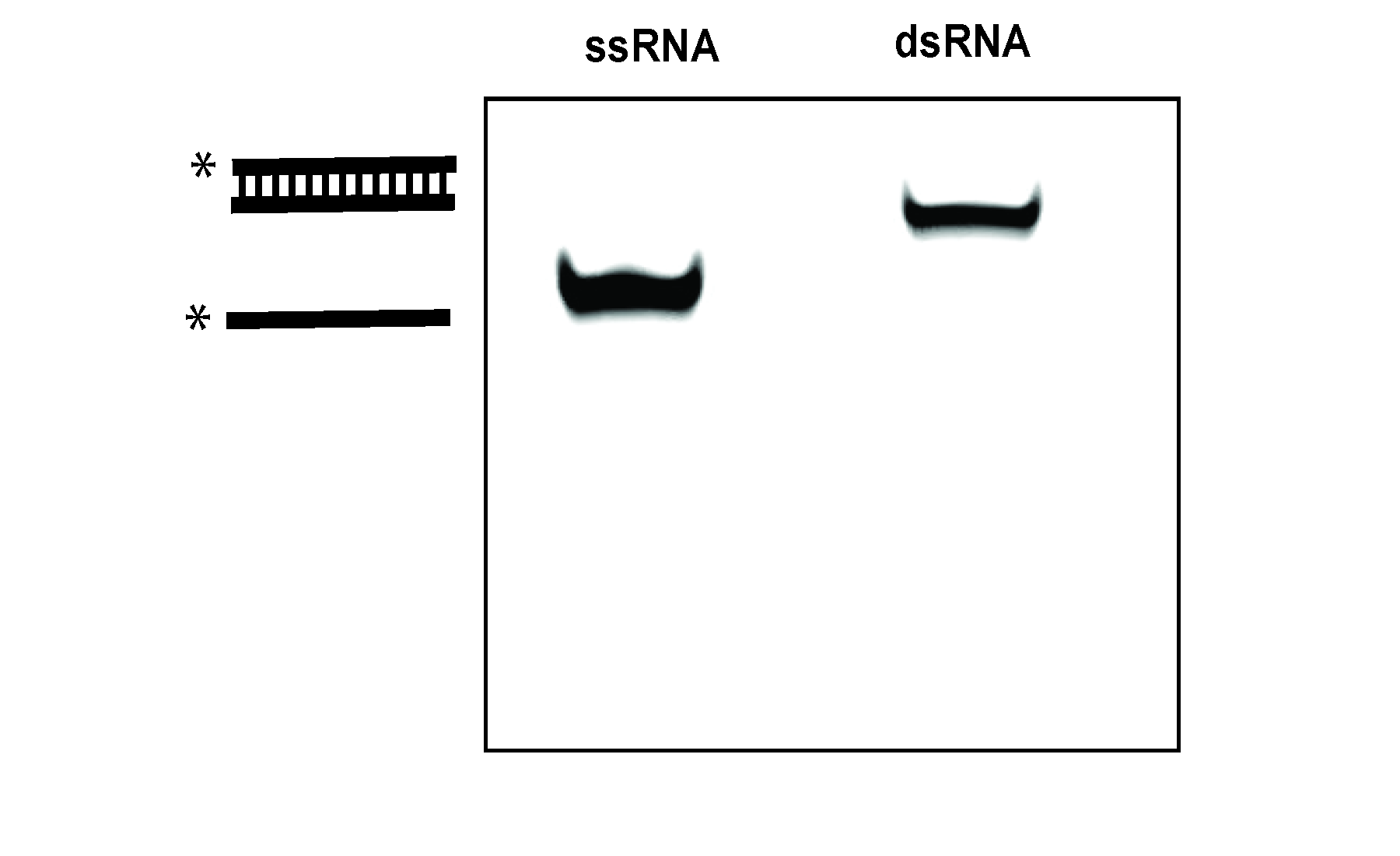

Supplement: S12 Fig — SDS-PAGE gel showing cleavage of MBP-2C at different time points with 250 nM of TEV protease used in the dsRNA unwinding assay at room temperature and 37°C and the corresponding quantitation of the cleavage. (TIF) [file ppat.1012388.s012.tif]

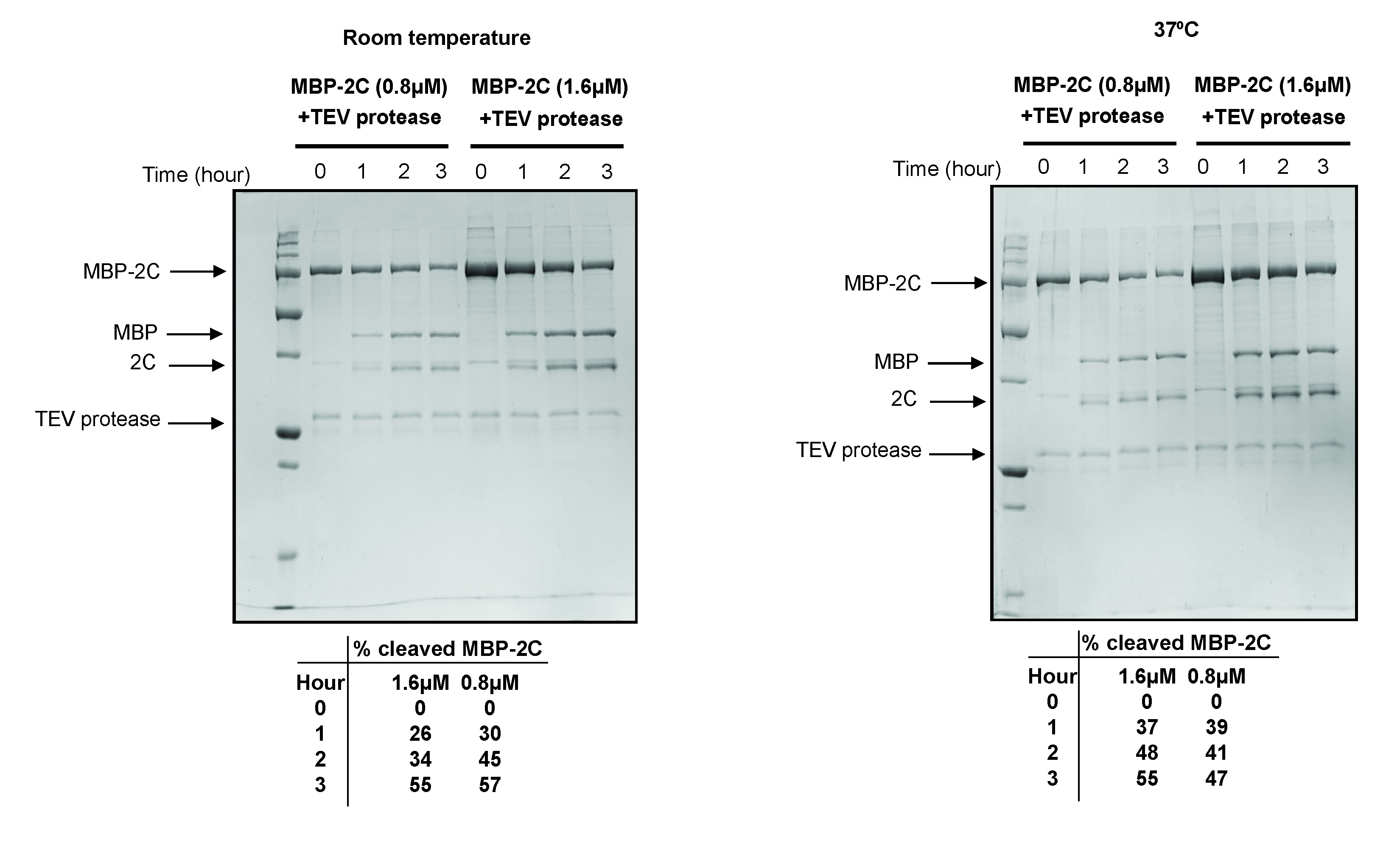

Supplement: S13 Fig — Ribonuclease activity of membrane-bound 2C is Mg2+-dependent but ATP-independent (A-B) Fluorescence scans of native polyacrylamide gel for dsRNA substrate with 5′ (A) and 3′ (B) overhang. The first two lanes of each gel contain ssRNA control and dsRNA substrate. The following lanes correspond to the last lane in Fig 6B and 6F, except that they have either Mg2+ or Mg2++ATP added. Representative gels of four independent replicates are shown. (C) Experiment as (A) but using ssRNA substrates fluorophore-labeled at 3′ or 5′ end as indicated. A representative of three independent replicates is shown. (D) The percentage of total RNA signal present as cleaved oligo is plotted for lanes of the gel in (C), and two independent replicates of the same experiment. Dots represent individual experiments, bars the average and error bars one standard deviation. Statistical significance by unpaired two-tailed Student’s t-test. ns: p>0.05, ****: p<0.0001 (TIF) [file ppat.1012388.s013.tif]

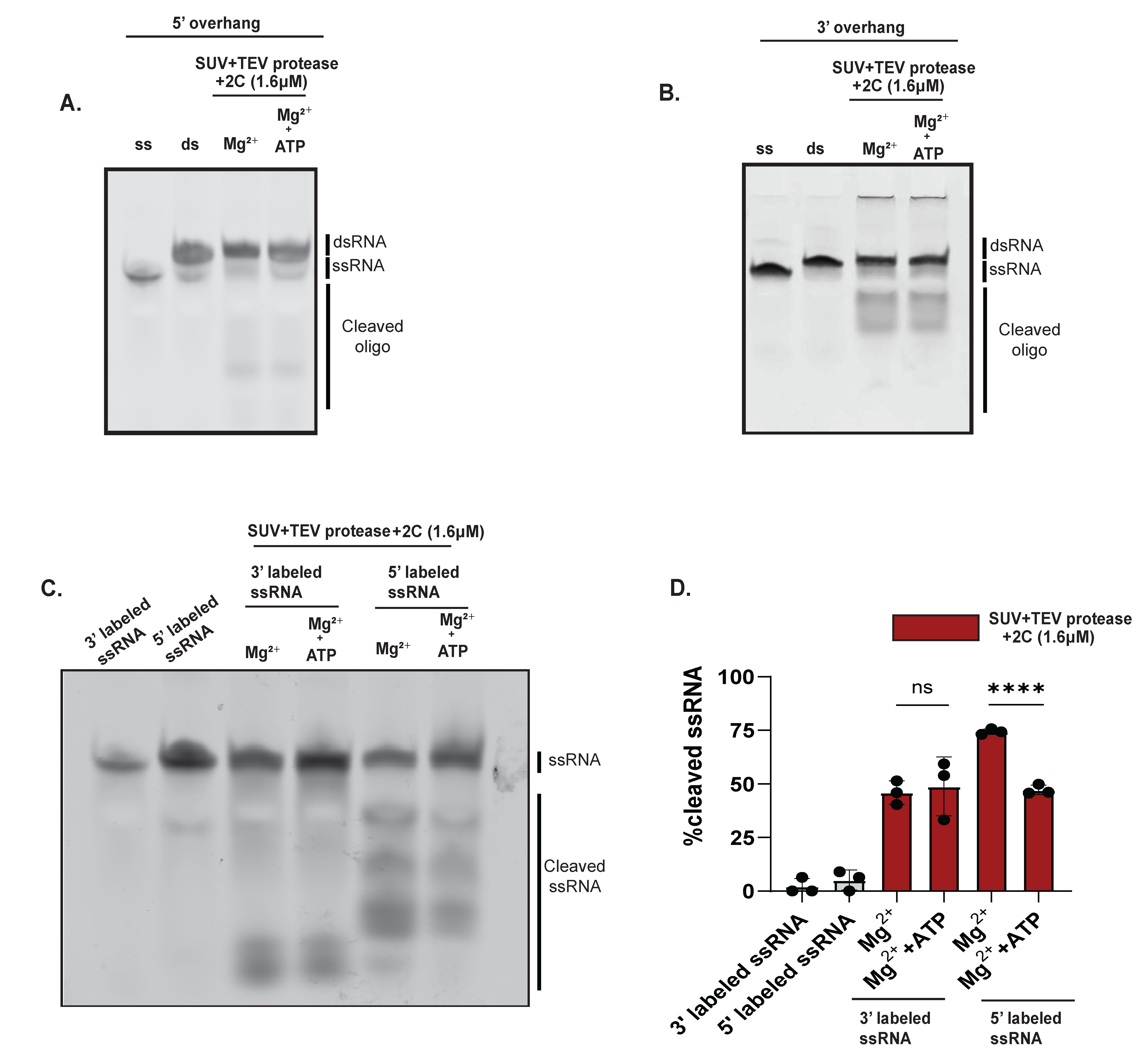

Supplement: S14 Fig — (A) SDS-PAGE gel showing 2 bands corresponding to TEV protease (B) Peptide coverage of TEV protease from in-gel digestion mass spectrometry of the excised bands from (A). (TIF) [file ppat.1012388.s014.tif]
