# Supplementary material for: In vitro reconstitution reveals membrane clustering and RNA recruitment by the enteroviral AAA+ ATPase 2C
Source: PLoS Pathog. 2024 Aug 5;20(8):e1012388. doi: 10.1371/journal.ppat.1012388 (PMC11326647; doi:10.1371/journal.ppat.1012388)
Supplement: S1 Table — (DOCX) [file ppat.1012388.s015.docx]

| **Oligo** | **Sequence 5**′**-3**′ | **Type** | **Label** |
| --- | --- | --- | --- |
| **1** | CCUCUAACCACAGUCUGAUC (20) | RNA | 5′ Tetrachlorofluorescein (TET) |
| **2** | GAUCAGACUGUGG UUAGAGG (20) | RNA | - |
| **3** | TGAATGCGGCTAATCCCAACCT (22) | DNA | - |
| **4** | AAAAAAAAAAAACCAGGCGACAUCAGCG (28) | RNA | 3′ Carboxyfluorescein  (FAM) |
| **5** | CCAGGCGACAUCAGCGAAAAAAAAAAAA(28) | RNA | 5′ Carboxyfluorescein  (FAM) |
| **6** | CGCUGAUGUCGCCUGG (16) | RNA | - |
| **7** | UGAAUGCGGCUAAUCCCAACCU (22) | RNA | - |
