# Supplementary material for: In vitro reconstitution reveals membrane clustering and RNA recruitment by the enteroviral AAA+ ATPase 2C
Source: PLoS Pathog. 2024 Aug 5;20(8):e1012388. doi: 10.1371/journal.ppat.1012388 (PMC11326647; doi:10.1371/journal.ppat.1012388)
Supplement: Attachment — Submitted filename: 140944_1_rebuttal_3057766_sfdfmh.pdf [file ppat.1012388.s018.pdf]

We would like to thank all reviewers for their detailed and constructive feedback, which substantially helped improve the manuscript. We apologise for the time taken for the revisions, which was partially due to the first author (successfully) writing and defending her PhD thesis in the same time frame. We would like to point out already here that, based on reviewers' feedback, main figure 6 is completely redone and the conclusions of this figure have changed substantially. We no longer suggest RNA chaperoning activity (it was identified as being due to the high concentration of TEV protease, in a control suggested by the reviewers). Instead, our refined assay conditions with lower TEV protease concentration identified ribonuclease activity of membrane-bound full-length 2C, which is consistent with a publication from 2022 (PMID: 35947700).

Reviewer #1 (Evidence, reproducibility and clarity (Required)):

Evidence, reproducibility, and clarity

Summary:

In this study by Shankar and colleagues, the authors aim to understand the structure and function of the enterovirus 2C protein, a putative viral helicase with AAA+ ATPase activity. Using poliovirus (as a model enterovirus) 2C, the author's propose the protein contains two amphipathic helices (AH1 and AH2) at the N-terminus that are divided by a conserved glycine. Using purified MBP-tagged 2C and N-terminal 2C truncations, their data suggests AH1 is primarily responsible for clustering at membranes, whilst AH2 is the main mediator of 2C oligmerisation and membrane binding. Furthermore, 2C was suggested to be able to recruit RNA to membranes, with a preference for dsRNA, and the author's data implies that the helicase activity of 2C is ATP-independent. Instead, the ATP activity appears to be required for 2C hexamer formation or chaperone activity. The manuscript is generally well written /presented and the author's present very interesting data which raises several questions, some of which require additional experimentation to help support the author's conclusions. Specific comments are as follows.

We thanks the reviewer for the overall positive assessment, as well as the specific comments below.

Major Comments:

1. The authors use four main constructs throughout the paper: full-length 2C, 2C with deletion of AH1 (ΔAH1), 2C with both AH1 and AH2 deleted (ΔMBP) and 2C with an extended N-terminal deletion. From this, the author's draw conclusions on the function of both AH1 and AH2. One of the author's main conclusions is that AH2 is the main mediator of 2C membrane association (e.g., in line 169). However, is it possible to conclude the relative importance of AH1 vs AH2 without testing a construct containing the deletion of AH2 only (ΔAH2)? This should be generated and used alongside this data to fully define the relative importance of AH1 and AH2 in these assay and remove the possibility that the deletion of AH1 changes the structure and/or function of AH2, which could also result in the observed differences.

This was a very good suggestion. We expressed and purified the ΔAH2 protein requested by the reviewer and characterized its oligomeric state as well as its membrane binding. It turns out, as suspected, that the ΔAH2 protein behaves very similarly to the ΔMBD protein (i.e. it does not form higher order oligomers and does not bind membranes). The changes in the manuscript due to this addition are many but can primarily be found in main figures 2-3 and their associated supplementary figures.

2. Previous structural predictions of 2C do not appear to have two separate AHs at the N-terminus. Are the AH1 and AH2 structures predicted to be formed in the context of the entire 2C protein, 2BC precursors and polyprotein? Are there structural approaches that could provide experimental evidence for two separate AH at the N-terminus?

This is a good point. Previous predictions were not that detailed, partially since they were done in the pre-alphafold era. Unfortunately, we cannot think of a tractable experimental method that could verify the split nature of the amphipathic helix in the only context that would matter: the protein bound to a membrane. A long-term goal would be in situ structures of full-length 2C on membranes using cryo-electron tomography, but our current sample and data sets are not sufficient for this. We added a mention of the long-term need for experimental structures of full-length 2C on lines 315-318 in the discussion.

3. Why are the 2C dimers (lines 137-138) not apparent on the mass photometry data presented (figure 2)?

Different constructs were measured by mas photometry and SEC-MALS. Also, the required concentration is 100-1000x lower for mass photometry which will affect a dynamic equilibrium in case the same construct were measured by the two methods.

4. It appeared that binding of ΔMBD-2C was better when POPS is in the membrane (line 174). What is the explanation for this and was this finding significant?

Well spotted. It may meat that 2C has a second, lower affinity membrane-binding site which is charge-dependent somewhere outside the MBD. We now added a mention of this in the discussion, lines 321-323.

5. From the author's data on lipid drop clustering they conclude ΔAH1 is more effective for clustering, however, the ΔAH1 construct produces pentamers not hexamers (from Figure 2). Is formation of hexamers related to or required for membrane clustering?

ΔAH1 is LESS effective at clustering, not more. As for the mention of pentamers in the original submission: we now think this was an unfortunate choice of words. The mass photometry data for 2C(ΔAH1) could more parsimoniously be interpreted as a mix of hexamers and other (unknown to us) smaller oligomers such as trimers. We have removed all mentions of pentamers.

6. The replicon data presented in Figure 7 should include a replication-defective control (e.g., polymerase mutant), in order to compare how defective in replication ΔAH1 and ΔMBP deletions are compared to a fully-defective construct. Likewise, deletion of ΔAH1 in this construct is likely to affect processing of the viral polyprotein where several previous studies with picornaviruses have demonstrated that the residues in the P2'-P4' positions can change cleavage efficiency (e.g., PMID: 2542331), or the structure of 2C, leading to the reduction of replication.

Thanks for these good comments. We made the polymerase-dead (GDD-to-GAA) replicon and remeasured it side by side with the 2C replicons. It has a similar luciferase activity indicating that no replication takes place in the 2C deletion replicons. This is shown in the new figure 7. As for the possibility or processing defects, we mentioned this in the original discussion and have now cited the reference suggested by the reviewer in this context (line 324).

7. How does the author's model of ATPase-independent helicase activity and an APT-dependent required RNA chaperone activity fit with 2 step model for RNA binding and ATPase activity suggested by Yeager et al (PMID: 36399514)?

Acting upon comments from other reviewers, we completely redid the "helicase assay" in the revised manuscript. It turns out that the ATP-independent unwinding activity in the original

submission was an artefact of the assay conditions (specifically, of the TEV protease at the higher concentration we used in the old assay). In our improved assay we neither see helicase activity nor ATP-independent RNA chaperoning activity.

Optional major comments that would increase the significance of the work:

All of the optional comments below are exceptionally interesting. But given the long time needed for the several major changes to this manuscript (e.g. the ΔAH2 protein characterization and reoptimisation of the helicase assay) we believe it is more sensible to address them in future studies, for which the 2C reconstitution system can be used.

8. The preference for dsRNA over ssRNA appears to be quite small (Figure 5d). In the context of a viral infection where ssRNA is likely to outnumber dsRNA at different times during infection is this preference physiologically relevant? In relation to this, what size stretch of dsRNA is required for preference, and could this correspond to cis-acting RNA structural elements, dsRNA as it escapes 3D polymerase or as part of the RF and RI forms (PMID: 9343205)? What is the proposed mechanism of how dsRNA outcompetes membrane tethering of 2C? OPTIONAL

9. The author's study has been conducted in the absence of other viral non-structural proteins. What is the physiological importance of the observations, such as membrane interaction/clustering or RNA binding when presented in the context of the other replication machinery. OPTIONAL

10. Do 2C monomers, dimers and hexamers have different functions in viral replication perhaps at different stages of replication and which of these forms are relevant during viral infection or can they all be detected during infection? Can any suggested separate functional arrangements be separated by genetic complementation experiments? OPTIONAL

Minor comments:

1. The author's appear to interchange between naming/nomenclature of the constructs which makes it confusing to follow (for example, ΔMBD is the same as 2C(41-329) likewise, 2C(Δ115) is sometimes called 2C(116-329)). It would be much easier to follow if the naming of constructs was consistent throughout (unless I am misunderstanding some subtlety in the difference between such constructs).

Thanks very much for spotting this. We have fixed it.

2. The author's suggest a pentamer arrangement for the ΔAH1 construct, however in the mass photometry data (figure 2D), a hexamer is indicated with the arrow. It would be helpful to change the label to indicate the size of the pentamer where this is being generated, not the hexamer.

As mentioned above, we think the "pentamer" designation of the original manuscript was unfortunate. It is more parsimonious to interpret this as a mix of states, hexamer and undefined snaller.

3. In most figures, data for full-length 2C, ΔAH1 and ΔMBP is shown. However data for ΔMBP is missing in Figure 4. Using ΔMBP may demonstrate even lower clustering, hinting that AH2 is also involved in this process.

Thanks for this comment. In our view, it can be derived from figure 3 (which shows lack of binding to PC/PE membranes) that the ΔMBD construct would not cluster membranes under the conditions of the assay (clustering requires concomitant binding to two membranes). We now describe our rationale for this on lines 220-222. However, we did include the ΔMBD protein in the new negative staining TEM supplementary figure where it and ΔAH2 show no signs of clustering (figure S10).

4. I think it would be better for normalise the data in the flotation experiments such that the percentage of 2C in the upper faction is presented as relative to the amount of lipid in the upper fraction (presented in Figure S4).

The change suggested by the reviewer would make it impossible to show the important no-liposome control (leftmost bar in Fig. 3C) in the same plot as the other measurements. We believe that would unnecessarily complicate the figure. Thus, we opted to keep the measurement that are normalised by lipid fluorescence in the supplementary figure. Instead, we now added another mention of this supplementary figure in the legend to main figure 3.

5. At several places (e.g., lines 232 and 272) the author's refer to "realistic systems". I think the term "physiologically relevant" might be more appropriate.

Agreed and changed throughout.

6. Line 237: I think "y" is a typo and should read "by".

Thanks. This text was reworked due to the major changes to figure 6.

Reviewer #1 (Significance (Required)):

Significance

I have limited expertise with structural biology but specialise my research on positive-sense RNA virus replication, structure and function. This research is of interest to a broad audience of researchers investigating many positive-sense RNA viruses, which extends beyond the viral family studied here. The work utilises novel techniques to begin to understand the specific roles of 2C in poliovirus replication. The author's data add important incremental new insight into recent studies on viral helicase proteins as referenced in the study, however, a key limitation is understanding the importance/relevance of their observations during a viral infection.

We thanks the reviewer for this positive and nuanced appraisal of our work.

Reviewer #2 (Evidence, reproducibility and clarity (Required)):

The authors present an alternative assay system to investigate picornavirus 2C, a protein that is tricky to analyze biochemically in its full length form because of an amphipathic helix at the N-terminus. Poliovirus 2C is expressed with an N-terminal MBP tag, a 50kD protein that helps with solubility as is commonly used for 2C investigations. A difference here is that liposomes are included to mimic membranes for 2C attachment. The key findings are that 2C induces clustering of of liposomes, that double stranded RNA binding by 2C impacts this clustering effect and that a free N-terminus (after cleavage of MBP by TEV protease) is needed for RNA binding and an ATP independent (ie non helicase) RNA duplex separation activity.

Major:

In the floatation assays in figure 3 the authors use a system where MBP-2C is fluorophore-labeled with ATTO488 on exposed cysteines. Poliovirus and other enterovirus 2C has a very well characterized zinc finger domain that has cysteines coordinating a zinc ion. Mutation experiments previously showed that these cysteines are necessary for viral replication and 2C stability. Have the authors controlled for disruption of the zinc finger domain by the labelling of cysteines with ATT0488 and checked if the protein remains folded?

We completely agree with the reviewer and apologise for the omission in the original submission. We have now included a Zn content measurement, which shows unchanged levels between labelled and unlabelled 2C protein (Figure S7). Also, we now in the revised manuscript explicitly describe our original reasoning for labelling on native cysteines: the presence of two cysteines which are not necessary for viral replication and which are more solvent exposed-exposed (and thus more likely to be labelled) in the crystal structure of the soluble fragment of 2C (lines 176-181).

In the analysis of the amphipathic helix, did the authors include membranes in their structural predictions o just the free helix? How does inclusion of membranes impact the predictions? In the predictions in Figure D, only 2 of 4 show a kink and there doesn't seem to be a correlation between those that predict a kink or not and whether the hydrophobic side is aligned in Figure S1.

Unfortunately, predicting a protein structure with the interacting membrane is beyond what is currently doable with protein prediction methods (one would have to combine protein structure predictions with molecular dynamics simulations including a membrane). Based on general principles of protein structure, it is likely that there is some flexibility around G17. Thus there may not be a single "kink angle" for any given virus, but we believe that the presence of the kink (and offset hydrophobic surfaces) for a number of viruses lends credibility and robustness to the observation. We added some descriptions of this thinking on lines 126-127.

Based on previous structures of 2C from different viruses the N-terminal amphipathic helix containing region is predicted to localize on one face of the predicted hexametric structure tethering 2C to the membrane. How does the authors hypothesized model explain 2C dependent clustering? is there evidence that 2C hexamers can oligomerize further into dodecamers for example, maintaining separate faces to enable N-terminal interaction with different membranes? What is the distance between the liposomes in figure 4 at the points of density attributed to 2C? How does this compare to the size of 2C determined in previous structural studies? Is it consistent with one hexamer/2 hexamers sitting on top of one another?

These are very interesting questions but we believe it is prudent to limit our speculation at this point. Eventually, we hope that larger data sets of cryo-electron tomography, coupled to subtomogram averaging, may provide a more definitive answer. What we managed to do with our current cryo-electron tomography data set is to estimate the volume of individual protein densities, and from the volume calculate an estimated molecular mass of the individual complexes seen in the tomograms. This correlates very well with 2C hexamers (new figure 4D).

In the Discussion lines 278-285 the authors suggest that having MBP attached may reflect the polyprotein condition. Can they make a construct with MBP-2B2C to examine interaction with liposomes and assess 2C function?

This is a highly relevant question, but the biochemistry of 2BC is even more challenging than 2C, and we are unfortunately nowhere near being able to work with purified 2BC at the moment.

Discussion lines 293-296, the possibility of two different populations of 2C, binding RNA or membranes cannot be excluded, there is much more 2C around late in infection that present in early infection- the model in figure 8 doesn't acknowledge/capture this.

We have changed the model figure such that more 2C is seen later, and the clustering function is also seen late in infection. The original discussion text referred to (which is unchanged) talks about a "preferential role in RNA replication and particle assembly at later time points" specifically for this reason. We hope the new figure 8 is better at conveying this message.

Discussion lines 313-317, the authors don't reference a study where a mutant of foot-and-mouth disease virus 2C lacking the n-terminal amphipathic helix that could bind but not hydrolyze ATP, hexamerized in the presence of RNA that seems pertinent here (PMID: 20507978).

Thanks for the suggestion. However, after the extensive changes we made to the revised to figure 6 based on excellent reviewer comments (essentially: the RNA chaperoning activity turned out to be an artefact, the improved assay shows no sign of RNA unwinding but instead of 2C-mediated ribonuclease activity), these sentence of the original discussion lost most of their context and we opted to remove them.

Some evidence of MBP-2C cleavage by TEV in the different assays used should be presented as this is a major focus of discussion and currently no gels show TEV cleavage is happening.

Thanks for the suggestion – we agree. We now show these in the new supplementary figures S5 and S12.

Reviewer #2 (Significance (Required)):

The work presents an additional methodology to investigate a a protein that has previously been difficult to study. The authors acknowledge that there is still a lot of 2C biology that remains to be discovered.

Thanks, we agree.

Reviewer #3 (Evidence, reproducibility and clarity (Required)):

The manuscript provides insights into the role of the N-terminus in membrane binding and its importance in the various functions of 2C.

Major issues

Line 103-119. Is this novel? I thought people had done a lot of bioinformatic analysis of PV 2C (especially Wimmer) who also did mutational work to analyse the importance of various amino acids in the N-terminal helix. I feel like the paper in general, and this section in particular, underplays the large body of work that has been done on the amphipathic helix by various groups.

We apologise if our original manuscript didn't sufficiently acknowledge previous work in the field. In the first sentence of the mentioned paragraph (now lines 112-113) , we did however cite several papers that have previously addressed the amphipathic nature of the N-terminus of 2C. We have now added two more references along the same line, and changed the wording in a way that we hope better bring across that the amphipathic nature per se has been studies before. We would be

happy to add more specific references if the reviewer has any suggestions. However, the rest of our analysis IS indeed novel for the following reasons: (i) we show that the amphipathic region is not a simple, single amphipathic helix, but instead has a conserved glycine (helix breaker/destabiliser residue) and two distinct amphipathic stretches before and after this region, (ii) we use alphafold2 (not available at the time of the earlier work) to provide the first reliable structural models of the membrane-binding domain. These models consistently, across several enterovirus 2C proteins, reveal that the hydrophobic surfaces of the first and second amphipathic regions, on either side of the conserved glycine 17, are offset from one another. This lends additional credibility to the distinct nature of these regions which have not previously been identified as such and which we also show in the biochemical assays to be functionally distinct. We have now also added a clarification to the Discussion that the N-terminus of 2C had previously been identified as its membrane-binding domain and we cite references for this. We hope that these changes will sufficiently acknowledge earlier work in the field while clearly pointing out the advance that our paper makes.

Line 132. Did you validate your column with known MW standards? The peak for full length and deltaAH1 look fairly standard for 2C, in that you have a mixture of species. Not sure you can say it is a hexamer when it is such a broad peak. C doesn't really help you too much since the counts at 400 (pentamer) and 480 (hexamer) are almost the same with quite large error bars. Like most people that have worked with 2C I think the best you can say is that you are making some kind of oligomerized 2C that includes hexamer, pentamer, etc. Why no dimer for MBP-2C and MBP-2C(delta AH1) when compared to the other constructs?

We did not calibrate the gel filtration column since the outcome would anyway be a more crude estimate of molecular mass than the mass photometry and SEC-MALS measurements. But we do agree with the reviewer on the broad mass photometry peaks. To address this experimentally, we compared the existing MBP-2C spectra to new recordings on apoferritin, a highly stable homomultimeric protein complex of a similar mass to aa MBP-2C hexamer. The apoferritin mass estimate is overlayed with the full-length MBP-2C in the new figure 2D and the corresponding supplementary figure S3. This indeed shows that the MBP-2C peak is broader, i.e. consistent with a mix of species which are predominantly but not only hexamers. We describe and discuss this on lines 145-149. As for the mention of pentamers in the original submission: we now think this was an unfortunate choice of words. The mass photometry data for 2C(ΔAH1) could more parsimoniously be interpreted as a mix of hexamers and other (unknown to us) smaller oligomers such as trimers. We have removed all mentions of pentamers.

Line 143. Does your data show that there are two amphipathic helices? Bioinformatics suggests it but your experiments just show the importance of the two areas in oligomerization, not that it is forming two helices.

We agree that the choice of words was not idea and have now changed it to "structure predictions indicate" (lines 162).

Figure S2. Your preps are still relatively dirty, which isn't ideal for biochemical assays. Especially lane 3, where you are looking at 50-60% purity. I don't want you to re-run experiments but I think you need to comment on the purity of the protein you are working with. Also I don't like that you removed the top and bottom of the SDS-PAGE. How much protein never entered the gel. Is there a big fat band at 20 kDa? You need to have the full gel here. Did you measure 260 nm of the preps as well to see if you had bound RNA to the 2C?

Thanks for the comment, we agree that our original submission lacked detail in the description of the protein purification. This is now addressed with the new figure S2 which shows size exclusion chromatograms of the fluorophore-labelled proteins (same chromatograms as in figure 2) and the corresponding uncropped gels imaged both in the stain-free channel (showing all

proteins) and in the fluorescence channel. The A260/A280 ratio measured for all proteins shows that they are free of nucleic acids at the point of imaging. The protein preps are not 100% homogeneous but we do believe that they are more than 50-60% pure.

Lines 170. Wasn't this done in the recent "An Amphipathic Alpha-Helix Domain from Poliovirus 2C Protein Tubulate Lipid Vesicles"? I don't see it referenced. What is novel about the current work when compared to that paper? Any differences?

Thanks for pointing this out. The referenced study worked with a synthesized, isolated peptide corresponding to AH2 (i.e. not with full protein). An amphipathic peptide outside the context of its protein cannot be expected to recapitulate the properties of the entire protein, e.g. since it is not spatially constrained in how it interactis with membranes. As one example (relating to the title of that paper) we don't see full-length 2C protein tubulating membranes the way the isolated peptide does. As for the reviewer's question about novelty, the paper mentioned does not identify the split nature of the amphipathic region, does not consider the role of AH1, does not characterise the membrane-binding properties of full-length 2C with respect to liposome membrane composition and size, does not identify and characterise the membrane clustering properties of 2C, nor its interactions with nucleic acid when bound to a membrane. However, we do agree that we should have cited the paper in our manuscript. We now cite it in the discussion, lines 320-321.

I'm surprised by the lack of electron microscopy (negative stain mostly) of both the oligomerized 2C and the various liposomes. I know the Carlson group is a microscopy group so why the lack of validation using electron microscopy of the various DLS experiments? I know you did cryo-ET for one of the constructs but I think negative stain electron microscopy of other constructs would be useful.

Thanks for the suggestion. As suggested, we have now expanded the analysis with negative staining EM of several more constructs studied by DLS. It can be found in the new supplementary figure S10.

Figure 4C. What evidence is there that this is 2C apart from you added it to the liposomes? It also comes back to the relative impurity of your protein prep. Could this be E.coli contamination?

Thanks for this comment. We have now added a new supplementary figure (S5) showing SDS-PAGE gels of the reactions used for flotation and DLS assays – which are identical to the cryo-ET samples. In addition, we estimated the molecular mass of the individual, putative 2C desities in the cryo-electron tomograms by measuring their volume. This analysis, which can be found in the new figure 4D, shows that the estimated mass of individual protein densities is consistent with a hexamer of full-length 2C. In addition, we mention in the discussion the long-term need to determine high-resolution structures of membrane-bound 2C using cryo-ET and subtomogram averaging (lines 315-318).

Figure 8. Is this model supported by the data in this paper? Your cryo-ET says that 2C is there but that isn't supported by any other data. How is the dsRNA protected from the innate immune system in this model? is it just sat out in the cytosol? How is the nascent ssRNA packeged into the capsid? Is there competition between the dsRNA and capsid for 2C binding (which your model suggests)? I know it sounds like I am being overly critical of the model but in my opinion there are still too many unanswered questions in the field to come up with a half decent model.

Thanks for this comment. We are the first to agree that our understanding of the roles of 2C is far from complete! We should have been more clear that the model figure represents some of the roles of 2C identified to date, and does not claim to be complete. However we do feel that a model figure serves a purpose of putting our findings into a context, and also providing testable hypotheses for future research . As for the question, some of the roles of 2C shown in the model figure (in

particular, particle assembly) are rather supported but earlier work of ourselves and others. We have now produced a new model figure and changed the figure legend to better reflect the incompleteness of the current understanding, and the origin of the different parts of the model figure. In addition, we extended the final paragraph of the discussion (which lists still-unknown aspects of 2C) with the reviewer's mention of dsRNA shielding from innate immunity (lines 374-375). The other aspects mentioned by the reviewer as not yet fully understood are already mentioned in that paragraph.

Minor issues

Lines 43-45: I feel like you underplay the success of the poliovirus vaccination program. Approximately 30 of WPV1 in 2022 and the full eradication of WPV2 and 3. Vaccine derived polio is still an issue but even that is relatively low compared to where the world was in the 1950s.

We agree that the previous wording was not ideal. We replaced it and added another recent reference - related to the type 2 vaccine switch (lines 47-49).

Line 66. I agree there are 11 individual proteins but I feel like this leaves out the fact that some of the uncleaved precursors appear to have some functions, for example 2BC.

Good point. We have now added a mention of 2BC and the fact that it has distinct functions to the introduction (lines 70-71). 2BC is also mentioned in the legend of the model figure (figure 8).

Line 56: LD needs to be defined.

Well spotted thanks. Since the abbreviation was not used anywhere else we opted to spell it out instead (line 59).

Line 75. I think you have misrepresented Xia et al here. They clearly say that in their study that they show helicase and chaperone activity. I never managed to repeat that work but you should still report what they claim. One major thing is that they used insect expressed protein, whereas most people (including myself and in the paper under review) use E.coli expressed protein. Do post translational modifications play an important role in function?

You are right that the reference to their paper for this statement was incorrect. We have now made this part of the introduction more explicit (lines 82-83) and we also in the new discussion mention the possibility of e.g. post-translational modifications affecting 2C helicase activity, under reference to Xia et al (lines 359-361)

Line 103. Need to make it clear here it is poliovirus 2C.

Thanks, we added it (line 112).

Line 135. I assume you mean kDa instead of uM?

It should actually be µM. It is the solution concentration at which the assay was performed. We added some words to clarify this (line 154).

Figure 3. What do you mean by "Only 2C"? Is that MBP-2C? Maybe I am reading the data wrong but adding TEV does nothing? How do you know TEV is removing the MBP? It looks like MBP-2C binds to the liposomes just the same as cleaved MBP-2C. I see in line 165 you acknowledge this. Could an alternative conclusion for line 168 be that MBP isn't being cleaved off but that AH2 is too small to be

exposed in that construct? Did you do that construct without MBP being cleaved? I think you need to confirm that MBP is being cleaved off.

Thanks for spotting this mistake. It should indeed be MBP-2C (in the absence of liposomes). We corrected figure 3. Also, in response to this comment and similar ones, we have now added a new supplementary figure showing SDS-PAGE gels of the reaction loaded onto flotation assays and DLS (figure S5). It shows that MBP-2C is cleaved.

Line 184. Is there a reason you use the 2019 paper as a reference instead of the far earlier Bienz et al papers? I'd suggest they are the seminal papers on 2C membrane association. Once again how is this work different from the recent "An Amphipathic Alpha-Helix Domain from Poliovirus 2C Protein Tubulate Lipid Vesicles" paper?

See our response above of the paper mentioned here (which we have now cited). As for why we cite the 2019 paper here: our statement pertains specifically to the contact sites between lipid droplets and replication organelles, not to the membrane binding of 2C per se. We have now added a more general mention of membrane remodelling by non-structural proteins in the introduction, where we cite on of the Bienz papers (lines 75-77).

Figure 5D. So only 1-3% of RNA is found in the upper fraction? Is that significant enough to say that dsRNA was recruited significantly more than ssRNA? How confident are you in your quantification of the starting amounts of RNA?

We agree that the fraction is low, however, the fluorescence signal is very clearly above background. We are thus confident in the measurement. The low percentage at the end of the experiment likely has a simple physico-chemical explanation: in a dynamic equilibrium in a density gradient, whatever RNA dissociates during the run will migrate away from the 2C-vesicle fraction and not be able to rebind. We still tried to address this concern by a complementary experiment where we used fluorescence anisotropy to measure binding of RNA to 2C on vesicles. While the measurements showed the same tendency, they curves were not clean enough to be published, which we think is due to the complex system with 2C bound to vesicles and clusters of vesicles. Still, in view of the relatively low percentage of measured recruitment we opted to adjust the paper title and the title of figure 5 (including the subheading related to figure 5) to put less emphasis on the dsRNA recruitment.

Line 223. Any idea why the MBP needs to be cleaved off? Clearly the MDB is accessible or it would not bind to the liposomes.

Since we have no data directly supporting this we prefer not to speculate in the paper. But one guess would be that the NTD of 2C, as implicated by previous publications, has a dual role in membrane binding and RNA binding. It may be that it can bind membrane while conjugated to MBP, but needs MBP to be removed in order to simultaneously bind membrane and RNA.

Line 237: missing "b" in "by"

Thanks. This paragraph was rewritten in the light of the changes to figure 6.

Figure 6. I don't fully understand the results here. Earlier you showed that the delta MBD didn't really bind SUV. So presumably it isn't really membrane bound. Why does it have similar activity to full-length MBP in your helicase assay if membrane is important? Did you do SUV and TEV protease only control?

We are very grateful to this reviewer (and others) for pointing out the need for a TEV control. When performing the control, we found that the TEV protease, at the high concentrations initially used, surprisingly had an artefactual RNA chaperone-like effect on its own. We then proceeded to titrate down the TEV protease concentration to the point where it no longer interfered. At this TEV protease concentration, although 2C was substantially cleaved (see the new supplementary figure S12), we could no longer detect an RNA chaperone activity. Thus, the contents of the new figure 6, and its conclusions, have been substantially changed. We now focused our attention on the remaining effect that 2C has on RNA: single-strand ribonuclease activity. These experiments were all conducted in the presence of RNase inhibitors, and the presence of Mg^{2+}-dependent ribonuclease activity parallels a recent publication that found this for truncated 2C from hepatitis A and several enteroviruses.

Line 257: "staring"?

Thanks, corrected. A staring glycine would indeed be something strange.

Line 336. Need to change the u to mu.

Thanks, corrected.

Any discussion on your observation in Figure 1D that EV71 and CVB3 don't appear to have AH1 and AH2 or do you think that the domains are conserved across the different viruses?

Thanks for bringing this up. Based on this and a comment from another reviewer, we have now clarified our thinking around this. Since the glycine will introduce some flexibility between AH1 and AH2, we cannot say from the single alphafold predictions that this is THE kink angle. The presence of the kink in the predictions of several MBDs lends more credibility to the robustness of the observation, but most importantly the hydrophobic surfaces in AH1 and AH2 are non-aligned for ALL sequences we looked at. This is now described on lines 126-128.

Table 1 (and possibly elsewhere): an apostrophe is not the prime symbol. 5' compared to 5′ .

Thanks, we corrected this throughout.

Line 702 "and" should be "an".

Thanks, corrected.

I couldn't open one of the movies (140844_0_supp_2820374_a2g272.avi).

Sorry to hear this, we will check the movie again.

Reviewer #3 (Significance (Required)):

Overall I liked the paper and is worth publishing. One of the issues in the 2C field is the difficulty in making pure 2C and carrying out in vitro assays that correlate with what is observed in the natural infection. I think this paper suffers from similar struggles with a 2C preparation that doesn't appear

that pure. I think it also suffers from not having 2C from a wild-type infection. I don't think that it is feasible to get that kind of 2C but by once again using a recombinant protein from E.coli we are left with another manuscript that provides conflicting evidence of the functions of 2C without a definitive answer. The experiments are well done, although are missing some controls and the manuscript is laid out in a logical manner and is relatively easy to follow.

We thanks the reviewer for these comments. We believe that we have now provided better information regarding the purification of the recombinant 2C protein, and we do think that the controls present in the original manuscript and the revised manuscript alleviate the concerns about lack of specificity. Of course, isolating 2C vesicles from wildtype infection would be another interesting way of approaching its function, but such an approach would come with its own set of challenges related e.g. to the presence of confounding host factors.

Reviewer #4 (Evidence, reproducibility and clarity (Required)):

This is an interesting manuscript that reports the development of an in vitro membrane assay for probing the biochemical functions of the enterovirus 2C protein. The technique is interesting because it can be applied to 2C proteins from other members of the picornavirus family, an important group of mammalian pathogens. It has the capacity to probe different functions (e.g. membrane clustering, ATPase activity, RNA-binding and manipulation activities).

Overall, the manuscript is well written and gives a clear account of the work undertaken. It adds insight to previous studies of enteroviral (and picornaviral) 2C proteins, providing confirmation of some earlier work in a more physiological context and some new insights, particularly into the membrane and RNA binding aspects of 2C.

That said, there are a number of places where some amendment of the claims made is required to provide a more precise statement of the findings of this work. These are listed below.

We thank the reviewer for this positive feedback on our work, as well as for the sprcific comments below.

Line 21 (Abstract) - The authors claim to have shown that a conserved glycine divides the N-terminal membrane-binding domain into 2 helices. I would suggest instead what they have produced are computational predictions that this is the case - some way short of an experimental demonstration. Sequence analysis predicts helical secondary structure in the N-terminus and indeed Alphafold2 also predicts a helical structure, but these predictions require experimental verification. The authors should therefore rewrite sections that claim to have shown the presence of 2 helices. In doing so, they should perhaps also comment on the fact that Alphafold2 does not predict 2 helices in this region for all enteroviruses (see Fig 1D). Moreover, the sequence analysis in Fig. S1 shows the presence of two Lys residues in the segment 17-38; it would be interesting for the reader to have these indicated in the figures showing the Alphafold2 prediction - do they in any way interrupt the hydrophobic face of the predicted helix?

Thanks very much for this comment, which is in line with what other reviewers also wrote. We agree, and changed the abstract sentence. We have also rewritten the manuscripts in several places to address the limits of structure predictions and the eventual need for an experimental structure of full-length membrane-bound 2C (lines 126-128 and 315-318).

Line 82 (Introduction) - The authors write that the membrane binding domain (MBD) of poliovirus has been shown to mediate hexamerisation, citing Adams et al (2009) - reference 43. However, that is not what this paper shows. Rather it provides evidence of aggregation of an MBP-2C fusion protein into forms that ranged from tetramer to octamer, but no evidence that these aggregates assume functional forms (e.g. the presumed hexameric ring structure characteristic of the AAA+ ATPase family to which 2C belongs). As far as I am aware the first demonstration of hexameric ring formation by a picornaviral 2C protein was for the 2C of foot-and-mouth disease virus (see Sweeney et al, JBC, 2010). Although this is not an enterovirus, this finding was later confirmed for Echovirus 30 (ref 51). I should declare an interest here: the Sweeney paper is from my lab. I will leave it to the editor and the authors to determine how to write a more precise account of the early observations of hexamerisation in picornaviral and enteroviral 2C proteins.

Thanks very much for this insightful comment. As a response to this and other similar comments, we are much more cautious about our wording in the revised manuscript (see also response to comment below. In the part of the introduction discussed here (now lines 89-91) we now use the original wording of the Adams paper ("oligomerization"). In the context of that new text we didn't feel that Sweeney et al paper was a suitable reference, but we now cite it in the later mention of 2C's oligomeric/hexameric state in the first part of the Results (lines 137-138).

Line 132 - the authors used mass photometry to investigate oligomeric forms of their MBP-2C constructs and state that for the full length 2C protein "the high-mass peak closely corresponds to a hexamer". While it is true that the peak shown in Fig 2C aligns with the expected MW for an MBP-2C hexamer, the peak is very broad, indicative of the presence of other oligomeric states with lower and higher numbers of monomers. This should be commented on. Indeed, the finding seems to echo the early findings of Adams et al (ref 43) with poliovirus MBP-2C.

Thanks for this comment, which was also made by another reviewer. We cite here what we replied to that reviewer

…we do agree with the reviewer on the broad mass photometry peaks. To address this experimentally, we compared the existing MBP-2C spectra to new recordings on apoferritin, a highly stable homomultimeric protein complex of a similar mass to aa MBP-2C hexamer. The apoferritin mass estimate is overlayed with the full-length MBP-2C in the new figure 2D and the corresponding supplementary figure S3. This indeed shows that the MBP-2C peak is broader, i.e. consistent with a mix of species which are predominantly but not only hexamers. We describe and discuss this on lines 145-149.

Line 143 - for the reasons given above, this summary paragraph represents too strong a statement of what has been observed.

We agree, and changed the paragraph. It now only refers to "oligomerization" (lines 162-164).

Line 197 - I note that the authors did not test the membrane clustering capabilities of the 2C(41-329) construct. Although the 2C(deltaAH1) construct had already shown a significant loss of activity, the shorter construct could still have been a useful control. I don't think it is necessary for this experiment to be done, but if the authors have a rationale for not performing the experiment, perhaps they could include it in a revised manuscript.

Thanks for the suggestion. The rationale is that a protein that doesn't bind a membrane in the first place will also not cluster them (an action that requires binding TWO membranes). We now

describe our reasoning on lines 220-222. Nevertheless, we did test these constructs in the new supplementary figure showing negative staining TEM (figure S10).

Line 223 - typo. I think you mean MBD.

Thanks! Corrected (now line 257).

Line 215 - the authors observed that the presence of ssDNA reduced membrane clustering and conclude that "nucleic acid binding partially outcompetes membrane tethering activity". Two things: (1) although I agree is it likely that this effect is due to binding of DNA to 2C, binding has not been demonstrated experimentally so the authors should be more careful in how they describe their result; (2) there is no data presented to show that RNA binding reduces membrane tethering so at best I think the conclusion has to be that the data are consistent with the notion that DNA binding reduces membrane tethering. It would of course be interesting to see the effects of RNA and I'm curious to know why the assay was not performed.

Thanks for the comment. The honest answer is that previous publications (primarily Yeager et al, NAR 2022) convinced us that the outcome should be near-identical with DNA, so we chose DNA oligos because they are cheaper and easier to work with. But we agree with the reviewer that RNA is of course more relevant. We now present a comparison at 5 µM of ssDNA and ssRNA, which in fact shows a slightly stronger effect on membrane clustering by RNA (figure 5C). In the light of this additional experiment, we feel that some of the text changes suggested by the reviewer may no longer be necessary.

Line 237 - typo: by, not y

Thanks. In the light of the extensive changes to figure 6 this text was removed.

Line 284 - the authors claim that 2C may only bind RNA after the N-terminus is liberated from 2B in infected cells, since cleavage of the MBP tag from their construct was needed for 2C to bind RNA in their in vitro assay. However, this does not automatically follow given the large structural differences between MBP and 2B and the fact that the authors have not tested the RNA binding capacity of a 2BC fusion protein. Their claim here is too strong and should be re-written.

We agree, and have added a discussion along the lines suggested by the reviewer (line 330-332).

Line 293 - The authors speculate that RNA binding might cause a shift between the membrane clustering activities and the role of the protein in RNA replication. However, since they have not shown that RNA binding reduces membrane clustering, this is too speculative.

In our revised manuscript we have studied the effect of RNA on membrane binding, thus we feel that this text is relevant in the context of the extended experiments.

Line 299-317 - within this discussion is the assumption that in their assay system enterovirus 2C adopts the ring-like hexameric structure typical of AAA+ ATPases. While I agree this may well be the case, it has not been demonstrated in this study so the authors should make clear they are making this assumption. The same applies to the legend of Fig 8.

This part of the discussion was extensively rewritten after our changes to figure 6. We now only refer to "hexamer" once in the corresponding part of the discussion, where we talk about structural models of hexamers produced by other groups who have crystallised fragments of 2C. There we believe we should refer to hexamers to accurately cite their work.

We are not sure what the reviewer is referring to when it comes to the legend for figure 8: the original legend had no reference to the oligomeric state of 2C. We have substantially changed figure 8 and its legend and the new figure and legend make no references to hexamers/oligomers.

Line 302 - the authors claim to have shown that 2C is 'selective' for dsRNA. I think at best they have shown a preference for binding dsRNA over ssRNA.

We changed the wording (line 349). We have also changed the title of the paper where we removed "double-stranded".

Line 313 - The sentence starting "A recent study..." needs a reference.

The revised discussion no longer contains this sentence.

Line 332 - the full sequence of the synthetic gene used in this study should be made available (e.g. as supplementary information or a deposited sequence with an accession number). This is a critical point before the paper can be published.

We will of course submit the sequences as supplementary data. Thanks for the reminder.

Line 362 - the authors should describe the likely points of attachment of fluorophores and comment on how this labelling might affect 2C function.

Thanks for the comment. In response to this and a similar comment from another reviewer, we discuss the likely conjugation site of the fluorophore (lines 175-181), and also (due to the proximity to the Zn finger) provide a new measurement showing that equal amounts of Zn can be detected in the labelled and unlabelled protein (figure S7).

Line 372 - Is a single protein standard (BSA) sufficient to calibrate the SEC-MALS system?

Yes, it is the recommended procedure (note that SEC-MALS is only dependent on scattering, not elution volumes etc).

Reviewer #4 (Significance (Required)):

As stated above this is an interesting study that presents findings from a novel assay. It will be of interest to picornavirologists and the wider community interested in the mechanisms of AAA+ ATPases.

We thanks the reviewer for this positive appraisal of our work.
